# The intestinal carrier status of *Enterococcus* spp. in children: clonal diversity and alterations in resistance phenotypes before and after admission to a pediatric intensive care unit

**DOI:** 10.1186/s12887-023-04238-0

**Published:** 2023-08-30

**Authors:** Fariba Shirvani, Romina Hassanzadeh, Bahareh Attaran, Ghazale Ghandchi, Nafiseh Abdollahi, Zari Gholinejad, Zahra Sheikhi, Azita Behzad, Fatemeh Fallah, Leila Azimi, Azam Safarkhani, Abdollah Karimi, Alireza Mahdavi, Shahnaz Armin, Roxana Mansour Ghanaiee, Sedigheh Rafiei Tabatabaei, Seyed Alireza Fahimzad, Masoud Alebouyeh

**Affiliations:** 1 Pediatric Infections Research Center, Research Institute for Children’s Health, Shahid Beheshti University of Medical Sciences, Tehran, Iran; 2 Department of Microbiology, Faculty of Biological Sciences, Alzahra University, Tehran, Iran; 3 Pediatric Intensive Care Unit, Mofid Children’s Hospital, Shahid Beheshti University of Medical Sciences, Tehran, Iran; 4 Pediatric Intensive Care Department, Mofid Children’s Hospital, Shahid Beheshti University of Medical Sciences, Tehran, Iran; 5 Anesthesiology Research Center, Shahid Beheshti University of Medical Sciences, Tehran, Iran

**Keywords:** Enterococcus, Pediatric Intensive Care Unit, Carriers, MDR, VRE, HLGR, Antimicrobial Resistance

## Abstract

**Background:**

This study aimed to investigate the intestinal carrier status of *Enterococcus* spp. among children in a pediatric intensive care unit (PICU) and reveal the role of hospitalization in the alteration of resistance phenotypes and clonal diversity of the isolates during admission and discharge periods.

**Methods:**

Two separate stool samples were collected from hospitalized patients in the pediatric intensive care unit at admission and discharge times. The culture was done, and *Enterococcus* species were tested for antimicrobial susceptibility and carriage of v*anA-D* gene subtypes. Random Amplified Polymorphic DNA (RAPD)-PCR was used for a phylogenetic study to check the homology of pairs of isolates.

**Results:**

The results showed carriage of Enterococci at admission, discharge, and at both time points in 31%, 28.7%, and 40.1% of the cases, respectively. High frequencies of the fecal *Enterococcus* isolates with vancomycin-resistance (VR, 32.6% and 41.9%), high-level of gentamicin-resistance (HLGR, 25.6% and 27.9%), and multi-drug resistance phenotypes (MDR, 48.8% and 65.1%) were detected at admission and discharge times, respectively. Resistance to vancomycin, ampicillin, and rifampicin was higher among *E. faecium*, but resistance to ciprofloxacin was higher in *E. faecalis* isolates. The increased length of hospital stay was correlated with the carriage of resistant strains to vancomycin, ampicillin, and ciprofloxacin. While the homology of the isolates was low among different patients during hospitalization, identical (9%) and similar (21%) RAPD-PCR patterns were detected between pairs of isolates from each patient.

**Conclusions:**

The high rate of intestinal carriage of VR, HLGR-, and MDR-Enterococci at admission and during hospitalization in the PICU, and the impact of increased length of hospital stay on the fecal carriage of the resistant strains show the importance of antibiotic stewardship programs to control their transmission and spread in children.

## Introduction

*Enterococcus faecalis* (*E. faecalis*) and *Enterococcus faecium* (*E. faecium*) are among numerous bacterial species that can be found in the gastrointestinal tract of animals and humans [1]. These species comprise 1% of the intestinal microbiota in healthy adults [2, 3]. They can serve as opportunistic pathogens and are known as the common cause of urinary tract infections, bacteremia, endocarditis, burn and surgical site wound infections, abdomen, and biliary infections [4].

With the emergence of multi-drug resistant (MDR)-Enterococci, these species become a therapeutic challenge both in the hospital and community-acquired infections [5]. Resistance to ampicillin, vancomycin, and high-level aminoglycosides, which are traditionally prescribed for *Enterococcus* infections, is the main cause of treatment failure in clinical settings [6, 7]. People who treated with these antibiotics for long periods, those with a history of hospitalization or surgical procedures, patients with indwelling medical devices, immunocompromised patients, and those with underlying diseases are at higher risk of infections with MDR-Enterococci.

The intestine of children is colonized with *Enterococcus* spp. early after birth [8, 9]. The colonizing strains could originate from the mother and the environment [1–3, 5]. The risk of intestinal colonization with MDR- and vancomycin-resistant Enterococci (VR*E*) seems to be higher in children in lower age groups and those with prolonged hospitalization, immunosuppression, low birth weight, and antibiotic intake [8, 10]. Although these risk factors showed an association with the colonization of the gastrointestinal tract by MDR- and VR *Enterococci* among the ICU admitted children, there is a lack of data about their primary colonization status in the intestine of outpatient children and changes that could occur after their admission during hospital stays. To investigate this correlation, we aimed to detect the colonization of *Enterococcus* species in stool samples of children in the PICU at admission and discharge times. Moreover, the associations of patients’ demographics, antibiotic consumption, underlying diseases, and length of PICU stays with changes in the patterns of antibiotic resistance, carriage of *van*A-D genes, and their phylogenetic types were investigated.

## Materials and methods

### Patients and samples

A cross-sectional study was conducted in the PICU of Mofid Children’s Hospital, a referral hospital for infectious and non-infectious diseases in children, from July 2018 to February 2020. The PICU has nineteen active beds that admit approximately 1,440 children annually. In this study, 712 PICU admitted patients were screened for carriage of Enterococci. Stool samples were collected from the patients after obtaining an informed consent form from their parents. Two samples were collected from all the patients at the time of admission and before discharge. Patients with lower than 48 h hospitalization, neutropenic patients, and those who didn’t provide the two samples at defined time points were excluded from the study. After transfer to the laboratory, a swab from the homogenized samples was used for inoculation of Blood agar medium containing 6.5% salt and BD BBL™ Enterococcosel™ Agar (BBL, USA). Grown colonies were examined biochemically. Demographic and clinical data of the patients were recorded using a questionnaire.

### Characterization of *Enterococcus* species

Polymerase chain reaction was used to characterize *E. faecalis* and *E. faecium* strains. Genomic DNA was extracted from the freshly grown colonies using an extraction kit (GeNet Bio Company, Daejeon, Korea; Cat. No, K-3000) according to the instructions of the kit manufacturer. The primers that were used to perform the polymerase chain reaction (PCR) are listed in Table 1.

**Table 1:** Nucleotide sequences of the primers that were used for amplification of target genes and typing in this study

Name of primer	Target gene	Sequence (5’→3’)	Size of product (bp)	Source
EA1 (F)	*vanA*	GGGAAAACGACAATTGC	732	[31]
EA2 (R)	*vanA*	GTACAATGCGGCCGTTA		
EB3 (F)	*vanB*	ACGGAATGGGAAGCCGA	647	[32]
EB4 (R)	*vanB*	TGCACCCGATTTCGTTC	647	
EC5 (F)	*vanC1/2*	ATGGATTGGTAYTKGTAT	815/827	[32]
EC8 (R)	*vanC1/2*	TAGCGGGAGTGMCYMGTAA	815/827	
ED1 (F)	*vanD*	TGTGGGATGCGATATTCAA	500	[32]
ED2 (R)	*vanD*	TGCAGCCAAGTATCCGGTAA	500	
DD13 (F)	*ddl* (*E. faecalis*)	CACCTGAAGAAACAGGC	475	[32]
DD3-2 (R)	*ddl* (*E. faecalis*)	ATGGCTACTTCAATTTCACG	475	
FAC1-1 (F)	*ddl* (*E. faecium*)	GAGTAAATCACTGAACGA	1091	[32]
FAC2-1 (R)	*ddl* (*E. faecalis*)	CGCTGATGGTATCGATTCAT	1091	
RAPD 1247	RAPD sequence	AAGAGCCCGT		[33]
RAPD 1283	RAPD sequence	GCGATCCCCA		[34]

K = G or T; M = A or C; Y = C or T

### Antimicrobial susceptibility testing

Fresh colonies of the *Enterococcus* isolates were used for antibiogram using the disc diffusion method, according to the Clinical and Laboratory Standard Institute guideline [11]. The antibiotics were selected from 9 different families, including ampicillin (Amp, 10 µg), vancomycin (Van, 30 µg), erythromycin (E, 15 µg), tetracycline (Tet, 30 µg), ciprofloxacin (Cip, 5 µg), rifampin (Rif, 5 µg), chloramphenicol (Chl, 30 µg), nitrofurantoin (Nit, 300 µg) and high-level gentamicin (HLG, 120 µg). *E. faecalis* reference strain ATCC 29,212 was used as quality control in this method.

### Molecular detection of *vanA*, *vanB*, *vanC*, *vanD* genes

To detect the frequency of *vanA*, *vanB*, *vanC*, *vanD* genes, multiplex-PCR using specific primers was used (Table 1). Extracted DNA was done as described above. The PCR mixture included 3.5 µl of double distilled water, 12.5 µl of Taq 2X master mix (Ampliqon, Cat No. A190303, Denmark), 0.5 µl of each primer (10 pmol/ml, Table 1), and 5 µl of DNA. Cycling conditions were done as follows: 1 cycle of initial denaturation at 94 ^o^C for 3 min, 35 cycles of initial denaturation at 94 ^o^C for 1 min, annealing at 54 ^o^C for 1 min, extension at 72 ^o^C for 1 min, and 1 cycle of final extension at 72 °C for 7 min.

#### RAPD typing and homology analysis

The randomly amplified polymorphic DNA (RAPD) typing method was used for the differentiation of pairs of the *Enterococcus* species isolated from each patient at the time of admission and before their discharge. Primer sequence AAGAGCCCGT was used to screen the strains following initial results obtained compared to primer GCGATCCCCA. PCR was done in the following conditions: 1 cycle of initial denaturation at 94 ^o^C for 4 min, 1 cycle of primary annealing at 36 ^o^C for 4 min, 1 cycle of primary extension at 72 ^o^C for 4 min, 40 cycles of initial denaturation at 94 ^o^C for 30 s, annealing at 36 ^o^C for 1 min, extension at 72 ^o^C for 2 min and 1 cycle of final extension at 72 °C for 10 min. The phylogenetic relationship of *Enterococcus* strains based on antimicrobial susceptibility patterns, carriage of *vanA-D* genes, and RAPD-PCR patterns was determined using NTSYS software, version 2.20. The strains with 100% homology were considered identical, while others with ≥ 95% were defined as related and similar strains.

### Data analysis

Statistical analysis was done by SPSS software version 23. To determine the relationship between antimicrobial susceptibility and underlying diseases, gender, age, history of previous hospitalization, and length of PICU stay, a student *t*-test was used. A *p*-value ≤ 0.05 was considered statistically significant.

## Results

### Patients’ demographics

Out of the patients admitted to PICU during the study period, colonization of *Enterococcus* spp. was screened in 132 pairs of samples from the patients at the admission and discharge times. Patients that did not provide either the primary or the secondary samples were excluded from the study. Results showed colonization in 71.2% (94/132) of the patients at the admission time and in 68.9% of them (91/132) at the discharge time. Considering the admission and discharge times for each patient, *Enterococcus* colonization was shown in 31% (41/132) of the cases just at the admission time, in 28.7% (38/132) just at the discharge time, and in 40.1% (53/132) of them at both time points. The patients belonged to ages ranging from < 1 y (58.1%), 1–3 y (16.3%), > 3–6 y (7%), > 6–10 y (14%), and > 10 y (4.7%). Hospitalization days in PICU varied from ≤ 3 days (39.5%), 4–7 days (34.9%), and ≥ 8 days (25.6%). No significant difference in the carriage of Enterococci was detected on admission between the patients with a history of hospitalization or underlying diseases in comparison to those without a history of hospitalization or underlying diseases (70.8% vs. 70.7% and 69.2% vs. 64.7%, respectively). Data about the administration of antibiotics, including cefazolin, carbapenems, glycopeptides, aminoglycosides, fluoroquinolones, imidazoles, macrolides, lincomycin, penicillins, ansamycins, and other beta-lactams, in the patients who concurrently carried *Enterococcus* spp. at the time of admission and discharge were recorded for these patients during the PICU stay (Table 2).

**Table 2:** Demographic data of the patients who carried *Enterococcus* spp. at admission and discharge times in a pediatric intensive care unit in Tehran

Variables	Frequency
Gender	
Female	34.9% (15/43)
Male	65.1% (28/43)
**Age**	
<1 year	58.1% (25/43)
1–3 years	16.3% (7/43)
4–6 years 7–10 years >10 years	7% (3/43) 14% (6/43) 4.7% (2/43)
**LOS**	
≤3 days	39.5% (17/43)
4–7 days	34.9% (15/43)
≥8 days	25.6% (11/43)
**Underlying diseases**	
Underlying disease	60.5% (26/43)
Non-underlying disease	39.5% (17/43)
**History of hospitalization**	
Yes	55.8% (24/43)
No	39.5% (17/43)
**Antibiotic therapy**	
Cephalosporin	72.1% (31/43)
Carbapenem	30.2% (13/43)
Glycopeptide	41.9% (18/43)
Aminoglycoside	9.3% (4/43)
Fluoroquinolone	4.7% (2/43)
Nitroimidazole	27.9% (12/43)
Macrolide	11.6% (5/43)
Lincosamide	7% (3/43)
Penicillin	2.3% (1/43)
Ansamycin	2.3% (1/43)
β-lactam combination agents	2.3% (1/43)

LOS; Length of stay

### Species diversity of Enterococci in the stool of children at the admission and discharge times

Our results showed the dominance of *E. faecalis* in the fecal samples of the patients at both the admission and discharge times (67.4% and 62.8%, respectively). *E. faecium*, as the second prevalent species, was detected in 18.6% and 32.6% of the entry and discharge samples, while the other *Enterococcus* species constituted 14% and 4.7% of them at the same periods. Changes in the colonized species during the hospitalization period were detected in 30.2% of the children. These changes included *E. faecium* to *E. faecalis* (2.3%), *E. faecium* to non-*Enterococcus* colonizer (2.3%), and non-*E. faecalis*/non-*E. faecium* species to *E. faecalis* and *E. faecium* (9.3% and 4.7%, respectively), *E. faecalis* to *E. faecium* (9.3%), and *E. faecalis* to other *Enterococcus* species (2.3%) (Table 3).

**Table 3:** Alteration of antibiotic resistance phenotypes among the carriers of *Enterococcus* species in the pediatric intensive care unit

Antibiotic Resistance Among the fecal Enterococci	Admission^a^. % (n = 43)	Discharge^a^. % (n = 43)	*p-value*	Days of hospitalization (Mean ± SD)			*p-value*	Antibiotic prescription in hospital	*p-value*
				≤ 3	4–7	≥ 8			
**Vancomycin** (30 µg)	32.6% (14/43)	41.9% (18/43)	0.50	22.2% (4)	33.3% (6)	44.4% (8)	**0.04**	Cephalosporin	0.01
								Glycopeptide	0.01
								Aminoglycoside	0.02
								Nitroimidazole	0.04
*E. faecalis*	20.7% (6)	22.2% (6)	1	33.3% (2)	33.3% (2)	33.3% (2)	0.37	-^b^	-
*E. faecium*	50% (4)	78.6% (11)	0.3	18.2% (2)	36.4% (4)	45.5% (5)	1	-	-
**Gentamicin** (120 µg)	25.6% (11/43)	27.9% (12/43)	1	41.7% (5)	16.7% (2)	41.7% (5)	0.20	Aminoglycoside	0.07
								Macrolide	0.02
*E. faecalis*	24.1% (7)	29.6% (8)	0.75	50% (4)	25% (2)	25% (2)	0.72	-	-
*E. faecium*	12.5% (1)	21.4% (3)	1	33.3% (1)	0% (0)	66.7% (2)	0.38	-	-
**Ampicillin** (10 µg)	41.9% (18/43)	48.8% (21/43)	0.66	23.8% (5)	33.3% (7)	42.9% (9)	**0.02**	Cephalosporin	0.04
								Glycopeptide	0.06
								Aminoglycoside	0.04
*E. faecalis*	27.6% (8)	25.9% (7)	1	28.6% (2)	42.9% (3)	28.6% (2)	0.31	-	-
*E. faecium*	75% (6)	85.7% (12)	0.60	16.7% (2)	33.3% (4)	50% (6)	0.47	-	-
**Chloramphenicol** (30 µg)	4.7% (2/43)	9.3% (4/43)	0.67	25% (1)	50% (2)	25% (1)	0.81	-	-
*E. faecalis*	6.9% (2)	11.1% (3)	0.66	33.3% (1)	33.3% (1)	33.3% (1)	0.53	-	-
*E. faecium*	0% (0)	7.1% (1)	1	0% (0)	100% (1)	0% (0)	0.58	-	-
**Tetracycline** (30 µg)	74.4% (32/43)	67.4% (29/43)	0.60	48.3% (14)	27.6% (8)	24.1% (7)	0.26	-	-
*E. faecalis*	75.9% (22)	66.7% (18 )	1	66.7% (12)	22.2% (4)	11.1% (2)	0	-	-
*E. faecium*	75% (6)	64.3% (9)	0.61	11.1% (1)	44.4% (4)	44.4% (4)	0.17	-	-
**Nitrofurantoin** (300 µg)	4.7% (2/43)	0% (0/43)	0.49	0% (0)	0% (0)	0% (0)	1	-	-
*E. faecalis*	3.4% (1)	0 (0%)	1	0% (0)	0% (0)	0% (0)	1	-	-
*E. faecium*	12.5% (1)	0 (0%)	0.38	0% (0)	0% (0)	0% (0)	1	-	-
**Erythromycin** (15 µg)	72.1% (31/43)	74.4% (32/43)	0.76	31.3% (10)	37.5% (12)	31.3% (10)	0.74	-	-
*E. faecalis*	65.5% (19)	63% (17)	1	41.2% (7)	41.2% (7)	17.6% (3)	1	-	-
*E. faecium*	87.5% (7)	92.9% (13)	1	15.4% (2)	38.5% (5)	46.2% (6)	1	-	-
**Ciprofloxacin** (5 µg)	41.9% (18/43)	58.1% (25/43)	0.25	28% (7)	32% (8)	40% (10)	**0.05**	-	-
*E. faecalis*	27.6% (8)	40.7% (11)	0.38	36.4% (4)	36.4% (4)	27.3% (3)	0.48	-	-
*E. faecium*	75% (6)	85.7% (12)	1	16.7% (2)	33.3% (4)	50% (6)	0.47	-	-
**Rifampicin** (5 µg)	46.5% (20/43)	58.1% (25/43)	0.35	32% (8)	32% (8)	36% (9)	0.37	-	-
*E. faecalis*	31% (9)	37% (10)	0.76	40% (4)	40% (4)	20% (2)	0.86	-	-
*E. faecium*	87.5% (7)	92.9% (13)	1	23.1% (3)	30.8% (4)	46.2% (6)	0.57	-	-
**MDR**	48.8% (21/43)	65.1% (28/43)	0.19	32.1% (9)	35.7% (10)	32.1% (9)	0.31	-	-
*E. faecalis*	37.9% (11)	51.9% (14)	0.42	42.9% (6)	35.7% (5)	21.4% (3)	0.76	-	-
*E. faecium*	87.5% (7)	85.7% (12)	1	16.7% (2)	41.7% (5)	41.7% (5)	0.67	-	-
**Common Resistance phenotypes**	Van/Amp/Tet/Cip/Rif/E/G 9.3% (4)	Van/Amp/Tet/Cip/Rif/E 14% (6)	-	-			-	-	-
	Van/Amp/Tet/Cip/Rif/E 7% (3)	Van/Amp/Tet/Cip/Rif/E/G 9.3% (4)							
	Amp/Tet/Cip/Rif/E 4.7% (2)	Van/Amp/Cip/Rif/E/G 7% (3)							
		Van/Amp/Cip/Rif/E 7% (3)							
		Amp/Tet/Cip/Rif/E 7% (3)							
		Tet/E/G 4.7% (2)							
		Tet/Cip/Chl/E 4.7% (2)							

^**a.**^ In this table, results of resistance phenotypes for species of *Enterococcus* other than *E. faecalis* and *E. faecium* are not presented in detail. The total frequency of antimicrobial resistance, regardless of the species name, is shown in front of the antibiotic name

^b^. (-), Not significant

### Frequency of fecal enterococci with VR and HLGR phenotypes among the children

The carriage of VR*E* among the children varied between 32.6% (14/43) and 41.9% (18/43) at the admission and discharge times, respectively. The patients also carried HLGR Enterococci in a frequency of 25.6% (11/43) and 27.9% (12/43) at the admission and discharge times, respectively. Details about the diversity in the resistance phenotypes to the antibiotics are shown in Tables 3 and 4. Our results showed no significant changes in the frequency of resistance to different classes of the antibiotics among the fecal Enterococci isolates in the children before and after PICU admission. Carriage of *vanA* was detected in 57.1% (8/14) and 94.4% (17/18) of the characterized VR*E* at the time of admission and discharge, respectively. *vanB-D* genes were not detected in any of the VR*E* strains.

**Table 4:** Diversity and frequency of clinically important resistance phenotypes of *Enterococcus* spp. in the stool samples of children at the admission and discharge times

Bacterial species	Primary stool ^a^ N= (n, %)	Secondary stool ^b^ N= (n, %)
*E. feacalis*	67.4% (29/43)	62.8% (27/43)
*E. faecium*	18.6% (8/43)	32.6% (14/43)
Other- *Enterococcus* spp.	14% (6/43)	4.7% (2/43)
*E. faecalis-VRE*	42.9% (6/14)	33.3% (6/18)
*E. faecium VRE*	28.6% (4/14)	61.1% (11/18)
*E. faecalis HLGR*	63.6% (7/11)	66.7% (8/12)
*E. facium HLGR*	9.1% (1/11)	25% (3/12)
*E. faecalis MDR*	52.4% (11/21)	50% (14/28)
*E. faecium MDR*	33.3% (7/21)	42.9% (12/28)

^a^Primary stool sample; the stool samples were collected during the admission

^b^Secondary stool sample; the stool samples were collected > 48 h after admission to PICU

^c^VRE, vancomycin resistance Enterococci; HLGR, high-level gentamicin resistance; MDR, Multi-drug resistant phenotype

### Alteration of the antibiotic resistance phenotypes during the hospitalization

The most common drug resistance phenotypes were Tet/E (8/43, 18/6%) and Van/Amp/Tet/Cip/Rif/E (6/43, 14%) at the admission and discharge times, respectively. The most common multi-drug resistance patterns were Van/Amp/Tet/Cip/Rif/E/G (4/43, 9.3%) and Van/Amp/Tet/Cip/Rif/E (6/43, 14%) at the admission and discharge times, respectively. While Van/Amp/Tet/Cip/Rif/E MDR pattern was detected in two isolates of *E. faecalis* at the admission time (2/29, 6/9%), no common MDR phenotypes among *E. faecium* isolates were detected at the time of admission. The patients showed an increase in common MDR patterns at the discharge time, mainly among *E. faecium* isolates with Van/Amp/Cip/Nit/Rif/E (3/14, 21.4%), Van/Amp/Tet/Cip/Rif/E (4/14, 28.6%), and Van/Amp/Tet/Cip/Rif/E/G (2/14, 7.4%) phenotypes (Table 3).

The prevalence of VR*E* strains showed a relationship with the prescription of cephalosporins (*p* value = 0.01, 12.9%, 4/31), glycopeptides (*p* value = 0.01, 27.8%, 5/18), aminoglycosides (*p* value = 0.02, 75%, 3/4) and nitroimidazoles (*p* value = 0.04, 16.7%, 2/12). Prescription of macrolides (*p* value = 0.02, 40%, 2/5) and aminoglycosides (*p* value = 0.07, 75%, 3/4) also showed a relationship with the colonization of HLGR Enterococci in the admitted children (Table 3).

### Phylogenetic relationship between the *Enterococcus* isolates among the patients

**Fig. 1:**
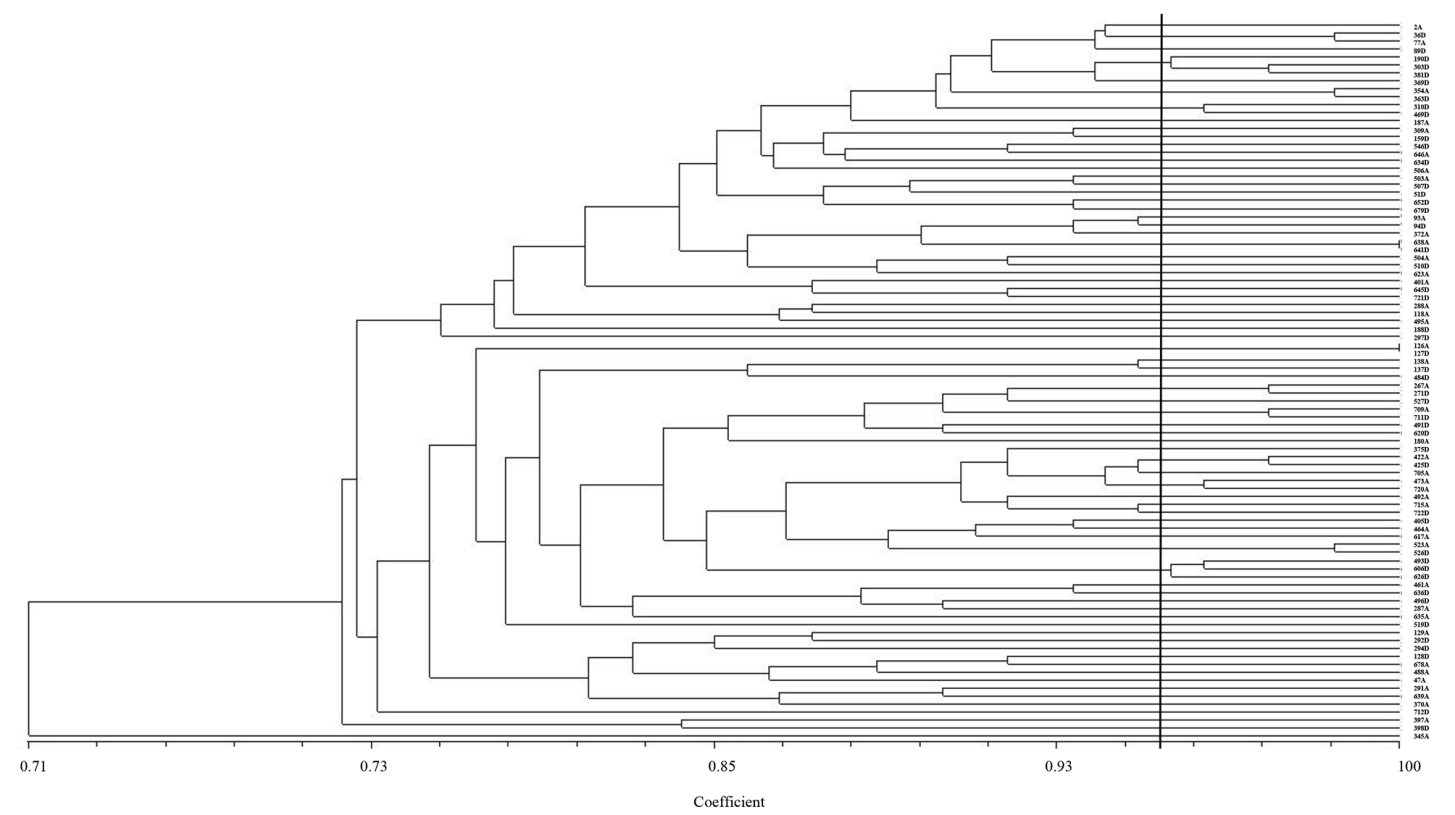
The relationship of *Enterococcus* strains based on the antimicrobial susceptibility- and RAPD-PCR patterns was determined using NTSyS software, version 2.20. *Enterococcus* strains with 100% homology were considered identical, while others with >  95% similarity defined as related strains. A and D letters refer to the strains isolated from the stool samples before and after admission to the PICU

In this study, a comparison of RAPD types and phenotypic characteristics of the *Enterococcus* isolates was made between pairs of the samples from each patient and among different patients in a PICU. Accordingly, the primary and secondary isolates from each patient showed identical, similar, or different origins in 7% (6/86), 21% (18/86), and 72% (62/86) of them, respectively (Fig. 1). Similarly, comparison of the isolates among different patients showed the identical, similar, or different clones of *Enterococcus* spp. in 4.6% (4/86), 15.1% (13/86) and 80.3% (69/86) of them, respectively. None of the patients with identical Enterococci clones were hospitalized during a similar time period, while 33.3% (6/18) of the Enterococci clones with similar patterns at each cluster were isolated from hospitalized patients during similar periods. History of hospitalization in the same hospital was not reported for any of the patients who carried identical or similar Enterococci clones.

## Discussion

In the current study, the fecal carriage of VR, HLGR, and MDR-Enterococci was shown in children both at the time of admission and on discharge. Moreover, their changes in antimicrobial resistance and phylogenetic patterns were confirmed during the PICU stay.

Enterococci usually become a problem in hospitalized patients who receive multiple courses of antibiotics [12]. VR and HLGR Enterococci can cause infection upon prolonged hospitalization and medical interventions [13, 14]. Prior history of antibiotics therapy and immunocompromised status are associated with Enterococcal infection in hospital settings. Compared with vancomycin-susceptible Enterococci, bacteremia due to VR*E* is considered an independent predictor of mortality and is correlated with a prolonged hospital stay in adults (4.5 days vs. < 1 day) [14]. Similarly, bacteremia by HLGR Enterococcal is associated with higher mortality compared with non-HLGR Enterococcal bacteremia [13]. There are few studies in children to present the impact of hospitalization on the alteration of Enterococci colonization. In the study by Endtz et al. in the Netherlands, the level of fecal transmission of *Enterococcus* was compared in the community and hospital, including PICU. They similarly reported a higher frequency of *Enterococcus* in stool samples of non-hospitalized people (80%) in compare to hospitalized patients (49%) [15]. In this study, VR*E* was detected in 2% and 2% of the non-hospitalized and hospitalized people, respectively. In a study by Hannaoui et al. in Morocco, *Enterococcus* colonization was determined in 70.3% of children’s stool samples (*E. faecium*, 55% and *E. faecalis*, 45%), and VR*E* was detected in 15.8% of them. All VR*E* strains in these children presented *vanA* resistance genotype [16]. A study in Turkey reported a positive culture rate of 30% in children at the admission time, which was lower than hospitalized cases (60%) [17]. *E. faecium* (70.4%), followed by *E. faecalis* (18.2%), *E. avium*, and *E. durans* were the dominant species. No VR*E* was detected in the samples upon admission and after hospitalization, and no phylogenetic relationship was reported among the hospitalized and outpatients children, although homology among the hospitalized children confirmed the spread of the identical strains in the hospital [17]. In a study in Tehran, which was conducted on stool samples from people in the community, *Enterococcus* was detected in all the samples [18]. While VR*E* and HLGR were not present among the isolates, molecular typing results confirmed the homology of the isolates in 61% of them. In the study in Ethiopia, the presence of *Enterococcus* was reported in 23% of the stool samples in hospitalized children (0–15 years old), which was much lower than that detected in the present study. VR*E* strains were found in 16.7% of the *E. faecium* isolates in this study [19].

A higher rate of VR*E* colonization in the gastrointestinal tract of people at the community level was reported by Adesida et al. in Nigeria (13.8%) [20]. In this study, *E. faecium* showed a similar frequency in compare to *E. faecalis*, but resistance to vancomycin was higher in *E. faecium* strains. Novais et al. reported VR*E* in 5% of their isolates, which is lower in comparison to our results [21]. Similarly, in the study of Barreto et al. on children aged 1–14 years in Portugal, 85.6% of children were colonized with *Enterococcus*; however, VR*E* was not detected in any of the investigated samples [22]. In Iran, in a study conducted by Farhadi and his colleagues in the NICU, the presence of VR*E* was confirmed in 42.2% of infants at the time of admission. All of these isolates were carriers of the *vanA* gene [23]. High colonization in these infants was significantly associated with antibiotic prescriptions for 7 days or more, referral from other hospitals, preterm birth, and low birth weight.

In the current study, there was a significant relationship between the duration of hospitalization and VR*E* colonization, implying that the amount of VR*E* colonization increased with the increase in the duration of hospitalization; however, no significant relationship was seen between the duration of hospitalization and HLGR and MDR colonization. The observed change in the colonization rate of patients hospitalized in ICU and its association with increased length of stay was previously shown in the study by Qiao and Xie et al. in China. In this study, while the rate of colonization of the gastrointestinal tract with VR*E* strains was 7.1% at the time of admission, this rate increased to 9% within 48 h of hospitalization and 16.5% and 18.9% after one week and thereafter at the time of discharge, respectively [24]. Similarly, in the study conducted by Jabbari-Shiade et al. and Karki et al., a significant relationship was observed between the length of hospitalization and VR*E* colonization (*p-value* < 0.001) [25, 26]. Contrary to these findings, such a relationship has not been confirmed in other studies [10, 27, 28]. While Viagappan and his colleagues did not find a significant relationship between the length of hospitalization and HLGR colonization, Mulin et al. reported a significant relationship between the length of hospitalization and HLGR colonization [29, 30].

In the case of the carriage of HLGR Enterococci in outpatients in compare to inpatients, the study of Kuzucu et al. showed 3% vs. 41% fecal carriage with the HLGR strains, respectively [17]. Similarly, Hannaoui et al. reported 2% HLGR and 88% MDR strains of Enterococci in human fecal samples at the community setting [16]. In the study of Gebrish et al., the frequency of MDR was 62.5%, which is similar to the result of our study [19].

Based on the data obtained from the patients’ files, the prescription of cephalosporins, glycopeptides, aminoglycosides, and nitroimidazole antibiotic families had a significant relationship with VR*E* colonization and administration of cephalosporins, macrolides, and aminoglycosides showed a significant relationship with HLGR colonization. However, administration of antibiotics didn’t show a significant relationship with intestinal colonization of MDR Enterococci. A study by Karki et al. showed that the administration of carbapenems and fluoroquinolones was significantly associated with VR*E* colonization [26]. The association of glycopeptides and cephalosporins administration with VR*E*-colonization was confirmed in a study in Iran [27] and India [10]; however, this link was not confirmed by the study of Kaveh et al. [28]. In the case of HLGR phenotype, Viagappan and colleagues found a significant association between cephalosporin administration and HLGR colonization, which is similar to our study [29].

This study has several limitations. Detection of *Enterococcus* species using the conventional culture method in the stool samples could not reflect its actual load within the intestinal microbiota. Follow-up studies at different time points during the hospital stay along with the microbiome analysis are needed to understand the impact of PICU admission and medications on the alteration of the microbial population in the intestine of children. Moreover, we were not able to compare the frequency of hospital-acquired infections caused by *Enterococcus* spp., mainly VR, HLGR, and MDR-Entrococci, between the carriers and non-carriers in the PICU. Indicating such a correlation is important to consider a preventive antimicrobial regimen for patients in the PICU.

## Conclusion

In the present study, carriage of MDR, VR, and HLGR *Enterococcus* species was shown in the stool samples of children either on admission or after hospitalization. Our results showed associations between the fecal carriage of VR and HLGR *Enterococcus* species and prolonged hospitalization and administration of broad-spectrum antibiotics in PICU admitted children. Although the hospital environment is considered the main source of transmission and intestinal colonization with MDR, VR, and HLGR Enterococci in children, non-hospital environments were detected as more likely sources of these clinically important bacteria in the studied population due to the detected diversity in phylogenetic patterns of the primary Enterococcus isolates on admission, mainly in children with no history of hospitalization. The detection of resistant strains in the intestinal tract of hospitalized children in the PICU following antibiotic therapy, which is considered a risk factor for hospital-acquired infection, emphasizes the importance of the implementation of infection control and antibiotic stewardship programs both at the hospital and community levels.

## Data Availability

The data of this study are available from the corresponding author upon request.

## References

[1] Dubin K, Pamer EG. Enterococci and their interactions with the intestinal microbiome. Microbiol Spectr. 2014;5. 10.1128/microbiolspec.BAD-0014-2016. 10.1128/microbiolspec.bad-0014-2016 PMC5691600 29125098

[2] Sghir A, Gramet G, Suau A, Rochet V, Pochart P, Dore J (2000). Quantification of bacterial groups within human fecal flora by oligonucleotide probe hybridization. Appl Environ Microbiol.

[3] Eckburg PB, Bik EM, Bernstein CN (2005). Diversity of the human intestinal microbial flora. Science.

[4] Zaheer R, Cook SR, Barbieri R (2020). Surveillance of Enterococcus spp. Reveals distinct species and antimicrobial resistance diversity across a one-health continuum. Sci Rep.

[5] Said HS, Abdelmegeed ES (2019). Emergence of multidrug resistance and extensive drug resistance among enterococcal clinical isolates in Egypt. Infec Drug rResist.

[6] Shirvani F, Behzad A, Abdollahi N (2021). Frequency and co-colonization of vancomycin-resistant Enterococci and Candida in ICU-hospitalized children. New Microbes New Infect.

[7] Arias CA, Contreras GA, Murray BE (2010). Management of multidrug-resistant enterococcal infections. Clin Microbiol Infect.

[8] Hufnagel M, Liese C, Loescher C (2007). Enterococcal colonization of infants in a neonatal intensive care unit: Associated predictors, risk factors and seasonal patterns. BMC Infect Dis.

[9] Fanaro S, Chierici R, Guerrini P, Vigi V (2003). Intestinal microflora in early infancy: composition and development. Acta Paediatr (Oslo Norway: 1992) Supplement.

[10] Amberpet R, Sistla S, Parija SC, Rameshkumar R (2018). Risk factors for intestinal colonization with vancomycin resistant Enterococci’ a prospective study in a level iii pediatric intensive care unit. J Lab Physicians.

[11] Wayne PCaLSI. ClSI. Performance standards for antimicrobial susceptibility testing. 28th ed. ClSI supplement m100. 2018.

[12] Gouliouris T, Warne B, Cartwright EJP (2018). Duration of exposure to multiple antibiotics is associated with increased risk of VR*E* bacteremia: a nested case-control study. J Antimicrob Chemother.

[13] Jang HC, Lee S, Song KH (2010). Clinical features, risk factors and outcomes of bacteremia due to Enterococci with high-level gentamicin resistance: comparison with bacteremia due to Enterococci without high-level gentamicin resistance. J Korean Med Sci.

[14] McNeil SA, Malani PN, Chenoweth CE (2006). Vancomycin-resistant enterococcal colonization and infection in liver transplant candidates and recipients: a prospective surveillance study. Clin Infect Dis.

[15] Endtz HP, van den Braak N, van Belkum A (1997). Fecal carriage of vancomycin-resistant enterococci in hospitalized patients and those living in the community in the Netherlands. J Clin Microbiol.

[16] Hannaoui I, Barguigua A, Serray B (2016). Intestinal carriage of vancomycin-resistant Enterococci in a community setting in Casablanca, Morocco. J Glob Antimicrob Resist.

[17] Kuzucu C, Cizmeci Z, Durmaz R, Durmaz B, Ozerol IH (2005). The prevalence of fecal colonization of Enterococci, the resistance of the isolates to ampicillin, vancomycin, and high-level aminoglycosides, and the clonal relationship among isolates. Microb drug Resist (Larchmont NY).

[18] Asadian M, Sadeghi J, Rastegar Lari A, Razavi S, Hasannejad Bibalan M, Talebi M (2016). Antimicrobial resistance pattern and genetic correlation in Enterococcus faecium isolated from healthy volunteers. Microb Pathog.

[19] Sisay Gebrish B, Tsegahun A (2019). Magnitude of drug-resistant *Enterococcus* species from intestinal tracts of hospitalized pediatric patients in Debreberhan referral hospital, Debreberhan, Ethiopia. IJRSMB.

[20] Adesida SA, Ezenta CC, Adagbada AO, Aladesokan AA, Coker AO (2017). Carriage of multidrug resistant Enterococcus faecium and Enterococcus faecalis among apparently healthy humans. Afr J Infect Dis.

[21] Novais C, Coque TM, Sousa JC, Peixe LV (2006). Antimicrobial resistance among faecal enterococci from healthy individuals in Portugal. Clin Microbiol Infect.

[22] Barreto A, Guimarães B, Radhouani H (2009). Detection of antibiotic resistant *E. coli* and *Enterococcus* spp. In stool of healthy growing children in Portugal. J Basic Microbiol.

[23] Farhadi R, Saffar MJ, Monfared FT, Larijani LV, Kenari SA, Charati JY (2022). Prevalence, risk factors, and molecular analysis of vancomycin-resistant Enterococci colonization in a referral neonatal intensive care unit: a prospective study in northern Iran. J Glob Antimicrob Resist.

[24] Qiao F, Xie Y (2011). Active surveillance for vancomycin-resistant Enterococci colonization in intensive care unit. BMC Proc.

[25] Jabbari-Shiade SM, Moniri R, Khorshidi A, Saba MA, Mousavi SGA, Salehi M (2013). Prevalence of vancomycin- resistant Enterococcus strains in fecal samples isolated from ICU patients in Kashan Shahid-Beheshti Hospital during 2011–2012. FEYZ.

[26] Karki S, Houston L, Land G (2012). Prevalence and risk factors for VRE colonisation in a tertiary hospital in Melbourne, Australia: a cross sectional study. Antimicrob Resist Infect Control.

[27] Askarian M, Afkhamzadeh R, Monabbati A, Daxboeck F, Assadian O (2008). Risk factors for rectal colonization with vancomycin-resistant Enterococci in Shiraz, Iran. Int J Infect Dis.

[28] Kaveh M, Bazargani A, Ramzi M, Sedigh Ebrahim-Saraie H, Heidari H (2016). Colonization rate and risk factors of vancomycin-resistant Enterococci among patients received hematopoietic stem cell transplantation in Shiraz, southern Iran. Int J Organ Transplant Med.

[29] Viagappan M, Holliman RE (1999). Risk factors for acquisition of gentamicin-resistant enterococcal infection: a case-controlled study. Postgrad Med J.

[30] Mulin B, Bailly P, Thouverez M (1999). Clinical and molecular epidemiology of hospital Enterococcus faecalis isolates in eastern France. Clin Microbiol Infect.

[31] Dutka-Malen S, Evers S, Courvalin P (1995). Detection of glycopeptide resistance genotypes and identification to the species level of clinically relevant Enterococci by PCR. J Clin Microbiol.

[32] Depardieu F, Perichon B, Courvalin P (2004). Detection of the van alphabet and identification of Enterococci and Staphylococci at the species level by multiplex PCR. J Clin Microbiol.

[33] Khan A, Das SC, Ramamurthy T (2002). Antibiotic resistance, virulence gene, and molecular profiles of Shiga toxin-producing *Escherichia coli* isolates from diverse sources in Calcutta, India. J Clin Microbiol.

[34] Ghalavand Z, Alebouyeh M, Ghanati K, Azimi L, Rashidan M (2020). Genetic relatedness of the Enterococcus faecalis isolates in stool and urine samples of patients with community-acquired urinary tract infection. Gut Pathogens.

